# Investigation of hypertension and type 2 diabetes as risk factors for dementia in the *All of Us* cohort

**DOI:** 10.1038/s41598-022-23353-z

**Published:** 2022-11-17

**Authors:** Shashwat Deepali Nagar, Priscilla Pemu, Jun Qian, Eric Boerwinkle, Mine Cicek, Cheryl R. Clark, Elizabeth Cohn, Kelly Gebo, Roxana Loperena, Kelsey Mayo, Stephen Mockrin, Lucila Ohno-Machado, Andrea H. Ramirez, Sheri Schully, Ashley Able, Ashley Green, Stephan Zuchner, SEEC Consortium, Priscilla E. Pemu, Alexander Quarshie, Kelley Carroll, Lawrence L. Sanders, Howard Mosby, Elizabeth I. Olorundare, Atuarra McCaslin, Chadrick Anderson, Andrea Pearson, Kelechi C. Igwe, Karunamuni Silva, Gwen Daugett, Jason McCray, Michael Prude, Cheryl Franklin, Stephan Zuchner, Olveen Carrasquillo, Rosario Isasi, Jacob L. McCauley, Jose G. Melo, Ana K. Riccio, Patrice Whitehead, Patricia Guzman, Christina Gladfelter, Rebecca Velez, Mario Saporta, Brandon Apagüeño, Lisa Abreu, Betsy Shenkman, Bill Hogan, Eileen Handberg, Jamie Hensley, Sonya White, Brittney Roth-Manning, Tona Mendoza, Alex Loiacono, Donny Weinbrenner, Mahmoud Enani, Ali Nouina, Michael E. Zwick, Tracie C. Rosser, Arshed A. Quyyumi, Theodore M. Johnson, Greg S. Martin, Alvaro Alonso, Tina-Ann Kerr Thompson, Nita Deshpande, H. Richard Johnston, Hina Ahmed, Letheshia Husbands, I. King Jordan, Robert Meller

**Affiliations:** 1 Georgia Institute of Technology, Atlanta, USA; 2 Morehouse School of Medicine, Atlanta, USA; 3 All of Us Demonstration Projects Subcommittee, National Institutes of Health, Bethesda, USA; 4 Vanderbilt University Medical Center, Nashville, USA; 5 The University of Texas Health Science Center at Houston, Houston, USA; 6 Mayo Clinic, Rochester, USA; 7 Brigham and Women’s Hospital, Boston, USA; 8 Hunter College, New York, USA; 9 Johns Hopkins University, Baltimore, USA; 10 National Institutes of Health, Bethesda, USA; 11 University of California, San Diego, USA; 12 University of Miami, Coral Gables, USA; 13 University of Florida, Gainesville, USA; 14 Emory University, Atlanta, USA

**Keywords:** Alzheimer's disease, Outcomes research, Cardiovascular biology, Diseases, Medical research

## Abstract

The World Health Organization recently defined hypertension and type 2 diabetes (T2D) as modifiable comorbidities leading to dementia and Alzheimer’s disease. In the United States (US), hypertension and T2D are health disparities, with higher prevalence seen for Black and Hispanic minority groups compared to the majority White population. We hypothesized that elevated prevalence of hypertension and T2D risk factors in Black and Hispanic groups may be associated with dementia disparities. We interrogated this hypothesis using a cross-sectional analysis of participant data from the *All of Us* (AoU) Research Program, a large observational cohort study of US residents. The specific objectives of our study were: (1) to compare the prevalence of dementia, hypertension, and T2D in the AoU cohort to previously reported prevalence values for the US population, (2) to investigate the association of hypertension, T2D, and race/ethnicity with dementia, and (3) to investigate whether race/ethnicity modify the association of hypertension and T2D with dementia. AoU participants were recruited from 2018 to 2019 as part of the initial project cohort (R2019Q4R3). Participants aged 40–80 with electronic health records and demographic data (age, sex, race, and ethnicity) were included for analysis, yielding a final cohort of 125,637 individuals. AoU participants show similar prevalence of hypertension (32.1%) and T2D (13.9%) compared to the US population (32.0% and 10.5%, respectively); however, the prevalence of dementia for AoU participants (0.44%) is an order of magnitude lower than seen for the US population (5%). AoU participants with dementia show a higher prevalence of hypertension (81.6% vs. 31.9%) and T2D (45.9% vs. 11.4%) compared to non-dementia participants. Dominance analysis of a multivariable logistic regression model with dementia as the outcome shows that hypertension, age, and T2D have the strongest associations with dementia. Hispanic was the only race/ethnicity group that showed a significant association with dementia, and the association of sex with dementia was non-significant. The association of T2D with dementia is likely explained by concurrent hypertension, since > 90% of participants with T2D also had hypertension. Black race and Hispanic ethnicity interact with hypertension, but not T2D, to increase the odds of dementia. This study underscores the utility of the AoU participant cohort to study disease prevalence and risk factors. We do notice a lower participation of aged minorities and participants with dementia, revealing an opportunity for targeted engagement. Our results indicate that targeting hypertension should be a priority for risk factor modifications to reduce dementia incidence.

## Introduction

Alzheimer’s disease and other dementias will affect > 30 million people in the US by 2040^1^, resulting in considerable stress and burden on patients, and caregivers. Alzheimer’s disease (the most common form of dementia) is characterized by a loss of cognitive capacity, and is common in the aging population^2^. While some familial/genetic cases have been described^2^, the cause of Alzheimer’s disease is still under debate, with anti-amyloid and anti-tau interventions currently predominating therapeutic approaches^3–5^. Understanding dementia risk factors may offer an opportunity to modify disease prior to symptom onset^1,6^. In 2019, the World Health Organization (WHO) identified hypertension and type 2 diabetes (T2D) as two modifiable risk factors for dementia^7,8^; although, the evidence in support of this conclusion has been challenged^9^.

Multiple studies show an increased prevalence of dementia in patients with hypertension^10,11^, or a strong effect of midlife hypertension on dementia prevalence^12^. Pharmacological control of blood pressure may reduce dementia incidence^8^. However, not all studies show a link between high blood pressure and Alzheimer’s disease forms of dementia^13^, and it is noted that blood pressure decreases with age^14^. The exact mechanism by which hypertension increases dementia risk is not yet clear, but it is thought that hypertension may contribute to vascular dementia via cerebrovasculature damage^15,16^.

T2D is also defined as a modifiable risk factor for Alzheimer’s disease and dementia^8^; although, prevention rather than treatment may be key^8^. In a pooled meta-analysis, T2D increased the risk of dementia in both men and women^17^, and two or more hypoglycemic episodes increased dementia risk^18^. Some studies suggest diabetes is a greater risk in vascular dementia versus Alzheimer’s disease^13,19,20^. Cognitive dysfunction is also reported in some T2D patients^21^. Studies report the link between T2D and dementias, especially common pathways associated with neurodegeneration and insulin resistance^22^, or linking molecular mechanisms induced in diabetes and AD pathogenesis^23^. A causal role for T2D, due to disease modifying therapy is less clear; one meta-analysis suggests a lower prevalence of dementia in patients administered metformin^24^, but this was not observed in other studies^18^. No diabetes modifying therapy has shown to be effective at controlling dementia^8^. Notwithstanding, several studies have linked common disease mechanisms of diabetes and dementia^25–27^.

Race and ethnicity have been implicated as a risk factor in dementia^1,8^. There is a higher prevalence (nearly twofold) of dementia in Black populations, compared to other racial groups and Asian populations appear to have a lower prevalence of dementia^1,28–30^. Some studies report a higher prevalence of dementia in the Hispanic population compared to the non-Hispanic population^28,30,31^. It is of note that cardiovascular risk factors for dementia are higher in Blacks; although, multivariable analyses including race and comorbidities is not typically included in such studies. We hypothesized that elevated prevalence of hypertension and T2D risk factors in Black and Hispanic groups may be associated with dementia disparities.

The *All of Us* (AoU) program was launched by the NIH in 2016 to create a longitudinal platform combining demographic, social, and biomedical data along with electronic health records^32^. The AoU Researcher Workbench was made available in 2019 for affiliated researchers to conduct approved AoU Demonstration Projects as part of data characterization and quality control prior to national release^32^. We investigated the relationship between dementia, hypertension, and T2D as an AoU Demonstration Project, in an effort to demonstrate the utility of the AoU Researcher Workbench for disease association studies. The objectives of our study were: (1) to compare the prevalence of dementia, hypertension, and T2D in the AoU cohort to previously reported prevalence values for the US population, (2) to investigate the association of hypertension, T2D, and race/ethnicity with dementia, and (3) to investigate whether race/ethnicity modify the association of hypertension and T2D with dementia. Our findings support the WHO position that hypertension in particular may be a modifiable risk factors for dementia, and we show that the association of hypertension with dementia varies among racial and ethnic groups.

## Results

### Demographic characteristics of the AoU cohort

**Figure 1:**
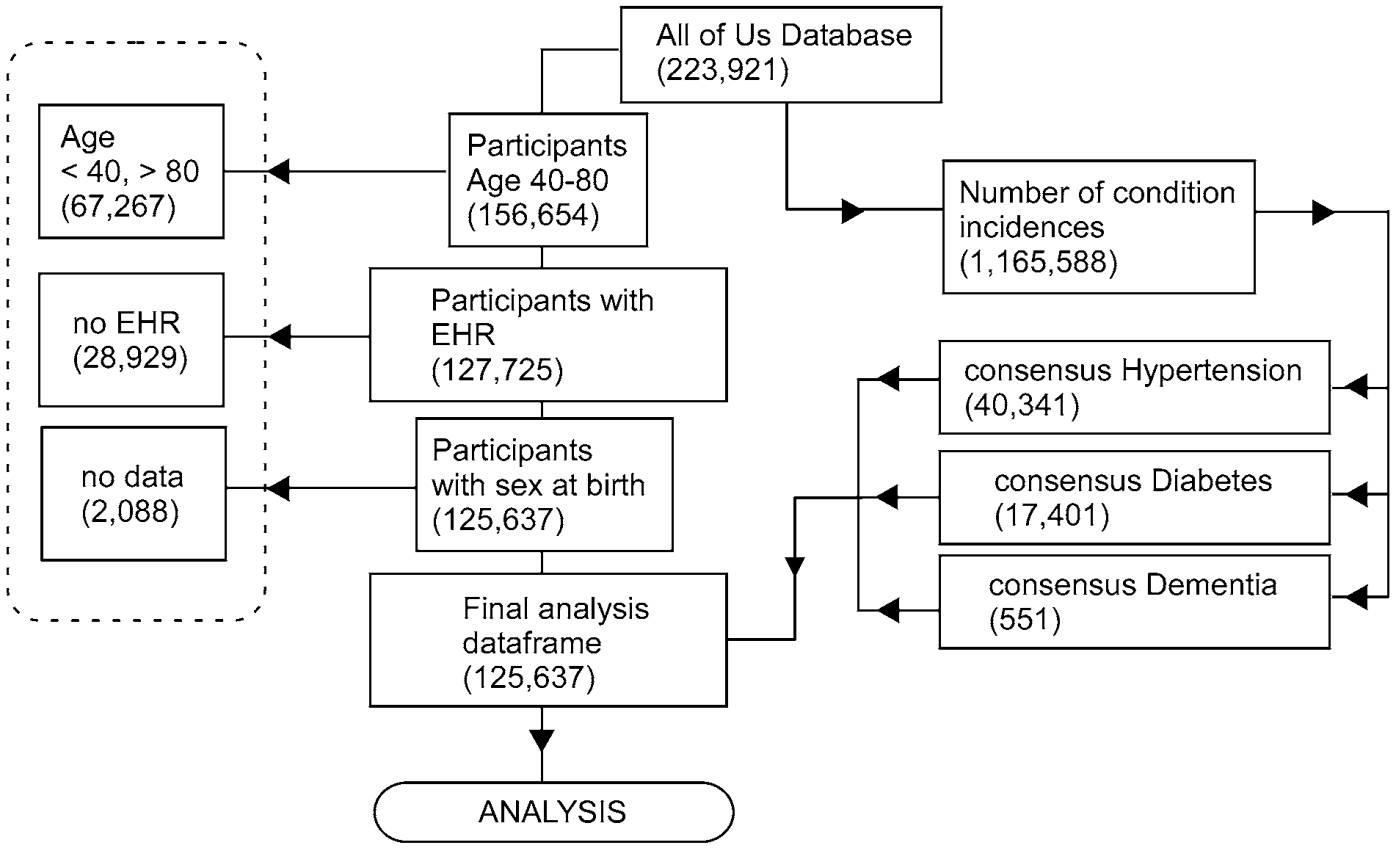
Overview of data inclusion/exclusion criteria from AoU Researcher Workbench to final participant cohort and analysis data frame. All steps were performed in R and are outlined in the Analysis workbook 01. Briefly, participant data were screened to exclude patients < 40 or > 80 years of age, and who did not have EHR data (defined as one visit or more to a health provider). Of the data containing medical conditions, we identified each unique participant ID with consensus diabetes, hypertension, or dementia (Supplementary Table 1) and annotated the data accordingly.

**Figure 2:**
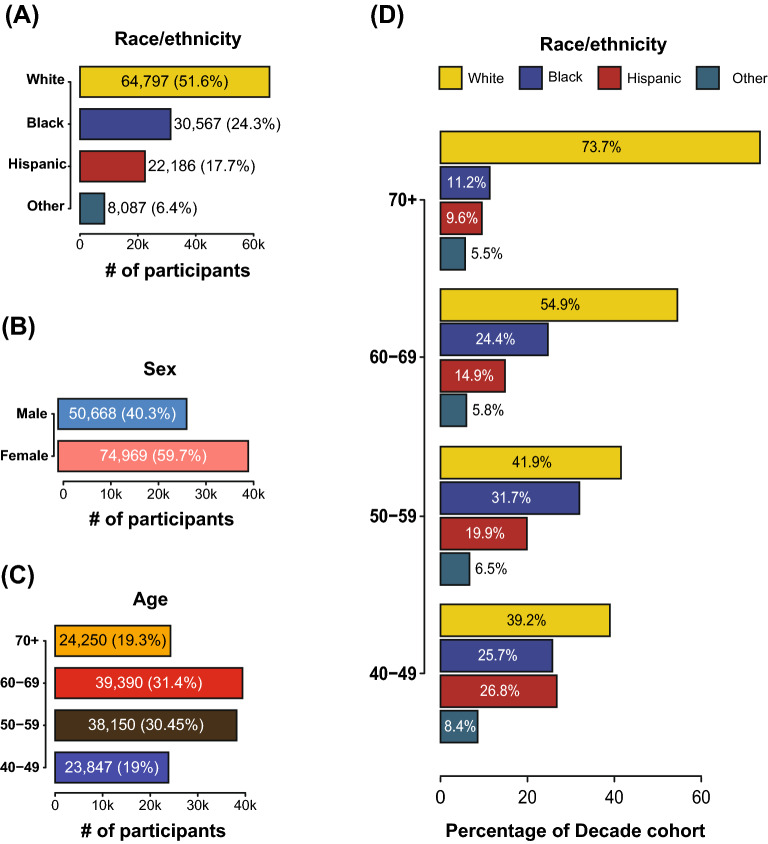
Demographic data for the AoU participant cohort. (**A**) Distribution of self-identified race/ethnicity (SIRE) labels in the study cohort. SIRE designations that were below the reportable minimum were merged with missing or generalized data. (**B**) Distributions of the sex at birth for participants who provided this information. (**C**) Age distribution of the participants included in this study cohort. Only participants above the age of 40 were selected for the study. Participants for whom this information was unavailable were not included in the study cohort. (**D**) Cohort proportions stratified by SIRE and age decade.

The AoU Researcher Workbench (at time of data freeze) contained data from 223,921 participants, of which 156,654 were aged between 40 and 80, 127,725 of these contained visit codes (EHR data), and when participants without information pertaining to sex at birth were excluded, a final cohort of 125,637 participants satisfied the inclusion criteria (Fig. 1). Demographic characteristics of the final AoU participant cohort for this study are shown in Table 1 and Fig. 2.

**Table 1:** Cohort description.

Characteristic (N = 125,637)	All participants in the cohort (N = 125,637)	Participants reported to have dementia (N = 551)	Participants reported to have hypertension (N = 40,341)	Participants reported to have type 2 diabetes (N = 17,401)
**Age—no. (Cohort share %)**				
40–49	23,847 (18.98)	< 20 (< 3.63)	4800 (11.90)	2248 (12.92)
50–59	38,150 (30.36)	65 (11.80)	11,083 (27.47)	5182 (29.78)
60–69	39,390 (31.35)	221 (40.11)	14,327 (35.51)	6207 (35.67)
70 +	24,250 (19.30)	248 (45.01)	10,131 (25.11)	3764 (21.63)
Mean age—year	60.13	68.03	62.49	61.67
**Race/ethnicity—no. (%)**				
White	64,797 (51.57)	294 (53.36)	20,058 (49.72)	6931 (39.83)
Black	30,567 (24.33)	103 (18.69)	10,924 (27.08)	5004 (28.76)
Hispanic	22,186 (17.66)	123 (22.32)	7027 (17.42)	4393 (25.25)
Other	8087 (6.44)	31 (5.63)	2332 (5.78)	1073 (6.17)
Female sex—no. (%)	74,969 (59.67)	330 (59.89)	24,267 (60.15)	10,434 (59.96)
**Condition—no. (%)**				
Dementia	551 (0.44)	–	451 (1.12)	250 (2.59)
Hypertension	40,341 (32.11)	451 (81.85)	–	14,895 (85.6)
Type 2 Diabetes	17,401 (13.85)	250 (45.37)	14,895 (36.92)	–

AoU participants self-identified their race and ethnicity (SIRE) upon project enrollment. 51.6% of participants identified as non-Hispanic White (‘White’ throughout the manuscript), 24.3% identified as non-Hispanic Black or African American (‘Black’ throughout the manuscript), and 17.7% identify as Hispanic of any race (‘Hispanic’ throughout the manuscript). A combined category of ‘Other’ was used to represent the remaining 6.4% of participants with various SIRE designations owing to AoU data size reporting rules (Fig. 2B). In the latest US Census data (July 2021), non-Hispanic Whites make up 59.3% of the population compared to 18.9% for Hispanic and 13.6% for Black. 40.3% of cohort participants were male and 59.7% were female (Fig. 2B).

Age affects the prevalence of dementia, hypertension, and T2D. 61.7% of participants are aged 50–69, with the remaining 38.3% under 50 or 70 and older (Fig. 2C). The prevalence of dementia in the AoU participants increases markedly for the 60 and over age group, whereas the prevalence of hypertension and T2D increase for the 50 and over age group (Table 1). SIRE groups show markedly different age distributions (Fig. 2D). The percent of White participants increases steadily with each decade, representing 73.7% of the 70 and over age group. In contrast, Black and Hispanic group participants are relatively over-represented at younger ages and at lower frequencies in the 60–69 and 70 and over age groups.

### Dementia, hypertension and T2D prevalence in the AoU cohort

**Figure 3:**
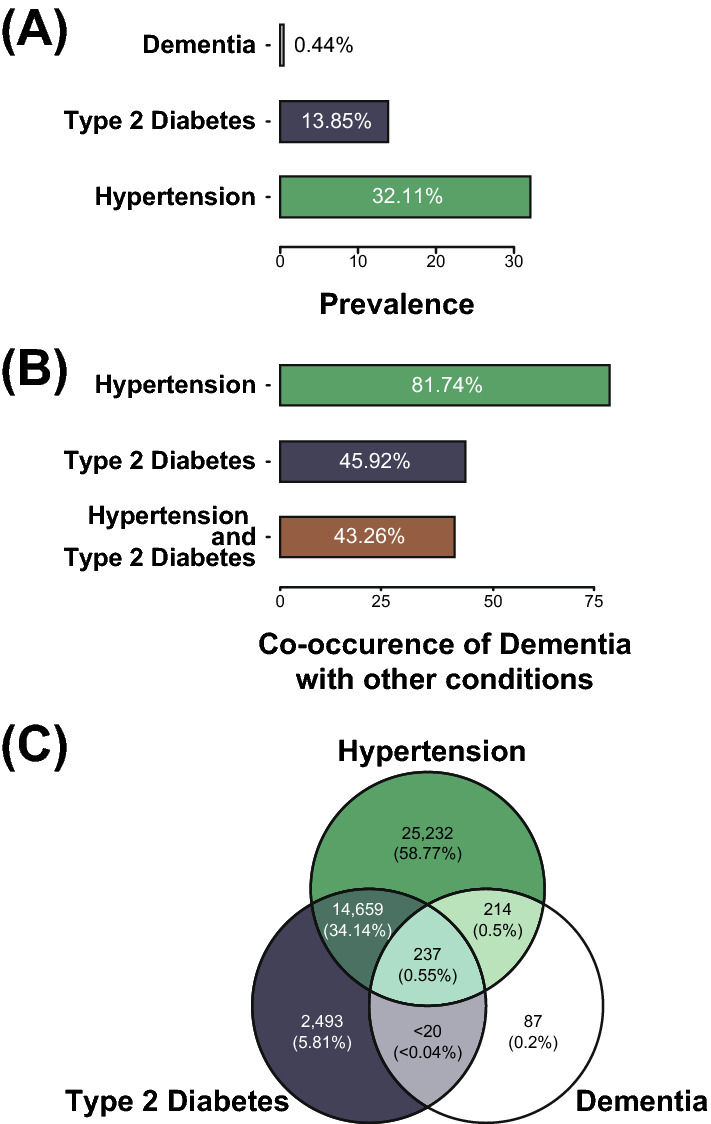
Prevalence for dementia along with hypertension and T2D comorbidities in the AoU participant cohort. (**A**) Prevalence of the three conditions under consideration: dementia, T2D, and hypertension. (**B**) Co-occurrence of dementia with hypertension and T2D. Each bar represents the percentage of participants with dementia who also have one or both of the comorbidities under investigation. (**C**) Venn diagram for the three conditions being studied. Values under the reporting minimum are represented as ‘< 20’.

551 cohort participants were diagnosed with dementia (0.44%), 40,341 with hypertension (32.1%), and 17,401 (13.9%) with T2D (Table 1; Fig. 3A). The prevalence of hypertension is similar to reported prevalence using the JNC7 criteria (32%), but see below^33^. Hypertension prevalence was highest at age 60 (35.5%). National estimates of T2D prevalence are 10.5% (rising to 26.8% at age > 65)^34^, which is similar to the values for the AoU cohort (Fig. 3A). In contrast, dementia prevalence in participants is lower than the national average (5 million/328 million US residents, 1.52%)^1^. We further investigated the difference in dementia rates by stratifying participants by age. While this increases the prevalence in ages > 60 (0.5%; Table 2), it was still below calculated age-adjusted prevalence rates (5%)^1^.

**Table 2:** Condition prevalence table.

Characteristic (N = 125,637)	Dementia	Hypertension	Type 2 diabetes
**Age—no. of cases (prevalence %)**			
40–49	< 20 (< 0.08)	4800 (20.13)	2248 (9.43)
50–59	65 (0.17)	11,083 (29.05)	5182 (13.58)
60–69	221 (0.56)	14,327 (36.37)	6207 (15.76)
70 +	248 (1.02)	10,131 (41.78)	3764 (15.52)
**Race/ethnicity—no. of cases (prevalence %)**			
White	294 (0.45)	20,058 (30.96)	6931 (10.70)
Black	103 (0.34)	10,924 (35.74)	5004 (16.37)
Hispanic	123 (0.55)	7027 (31.76)	4393 (19.80)
Other	31 (0.38)	2332 (28.84)	1073 (13.27)
**Female sex—no. of cases (prevalence %)**	330 (0.44)	24,267 (32.37)	10,434 (13.92)

Black (35.7%) and Hispanic (31.8%) participants showed higher prevalence of hypertension than White participants (31.0%) (Table 2). The AoU participant hypertension prevalence values are lower than the US population hypertension prevalence values published from the NHANES studies^35^. Hispanic (19.8%) and Black (16.4%) participants showed higher prevalence of T2D than White participants (10.7%) (Table 2), compared to national statistics of 12.5, 11.7, and 7.5%, respectively^34^*.* Finally, the prevalence of dementia was highest in Hispanic participants (0.55%) compared to White (0.45%) and Black participants (0.34%) (Table 2).

### Dementia, hypertension, and T2D comorbidities in the AoU cohort

We investigated the comorbidity prevalence for dementia, hypertension, and T2D. The prevalence of hypertension in participants with dementia was 81.6%, whereas the prevalence in participants without dementia was 31.9% (OR = 9.62, 95% CI 7.78–12.01, *X*^*2*^ = 625, df = 1, p < 2.2e-16) (Table 1; Fig. 3B). The prevalence of T2D in participants with dementia was 45.9%, whereas the prevalence in participants without dementia was 11.4% (OR = 5.23, 95% CI 4.42–6.18, *X*^2^ = 458, df = 1, p < 2.2e-16) (Table 1; Fig. 3B). Hypertension and T2D are highly comorbid for participants with dementia; > 90% of dementia participants who have T2D also have hypertension (Fig. 3C).

### Dementia risk factor associations and interactions

**Figure 4:**
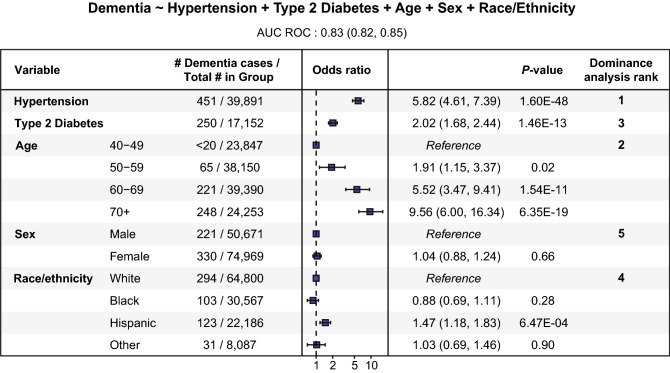
Modeling dementia status using hypertension, T2D, age, sex, and SIRE. Forest plot with the odds ratios and 95% confidence interval along with statistical significance for each of the predictor variables used to model dementia (outcome) status. The last column shows the relative importance of the variables obtained using dominance analysis. The logistic regression model specification is shown above the figure and the AUC score for the model is reported under the formulation of the model.

Multivariable logistic regression was used to model associations between dementia (the outcome) and hypertension, T2D, age, sex, and SIRE (the predictors), with dominance analysis used to compare the relative importance of the predictors (Fig. 4 and Supplementary Data 1). Hypertension showed the strongest association with dementia (OR = 5.8, 95% CI 4.6–7.4, p = 1.6e−48), followed by age (OR = 5.5, 95% CI 3.5–9.4, p = 1.5e−11) and T2D (OR = 2.0, 95% CI 1.7–2.4, p = 1.5e−13). Hispanic was the only SIRE category that showed a significant association with dementia (OR = 1.5, 95% CI 1.2–1.8, p = 6.5e−4), and sex was not significantly associated with dementia.

Interaction analysis was used to assess whether the associations of hypertension and T2D with dementia are modified by participants’ SIRE. The Cochran–Mantel–Haenszel test showed a significant interaction between hypertension and SIRE with respect to their association with dementia (M^2^ = 272.8, df = 3, p < 2.2e-16). In other words, the association of hypertension with dementia differed according to participants SIRE. Black participants showed a tenfold increase in dementia prevalence for hypertensive versus non-hypertensive participants (0.31% vs. 0.02%), whereas Hispanic participants showed a eightfold increase (0.48% vs 0.06%), and White participants showed a threefold increase (0.34% vs. 0.11%).

A significant interaction was also detected between T2D and SIRE using the Cochran–Mantel–Haenszel test (M^2^ = 1365, df = 3, p < 2.2e−16). The magnitude of this interaction was substantially lower than seen for hypertension. Hispanic participants showed a 1.6-fold increase in dementia prevalence for T2D versus non-T2D participants (0.34% vs. 0.21%), Black participants showed a 1.2-fold increase (0.18% vs. 0.15%), and White participants showed 1.9-fold lower dementia prevalence in T2D versus non-T2D participants (0.15% vs. 0.29%).

Interaction analysis was also performed by adding hypertension-SIRE and T2D-SIRE interaction terms to the dementia multivariable logistic regression model (Supplementary Data 1). This approach confirmed significant interactions of hypertension with Black (β = 1.3, se = 0.4, p = 3.8e−3) and Hispanic (β = 0.7, se = 0.4, p = 4.6e−2) groups but did not show any significant T2D-SIRE interactions. This is consistent with the lower magnitude of dementia prevalence in T2D versus non-T2D participants compared to the large differences seen for hypertensive versus non-hypertensive participants.

## Discussion

Here we report the results of an AoU Demonstration Project investigating the relationship between dementia, hypertension, and T2D along with participant demographic characteristics, race and ethnicity in particular. The AoU Research Program is a large an opt-in observational cohort study in the US, whereby participants agree to share electronic health record data and provide biospecimens for molecular analysis^36^. Participation is voluntary, and participant recruitment is targeted to enhance the inclusion of minority population underrepresented in biomedical research. As a result, the proportion of minority SIRE category participants appear higher than national census estimates. AoU Demonstration Projects were intended as a means to prototype and validate the Researcher Workbench platform and to assess the utility of the cohort for disease association studies; Demonstration Projects were not necessarily intended to generate novel findings.

We noticed a relatively similar prevalence of diabetes and hypertension in our 40–80 year old cohort, compared to reported national averages. The Centers for Disease Control and Prevention (CDC) reports a prevalence of hypertension at 33% of the population, while the US Department of Health and Human Services (HHS) statistics report 45% of the US population with hypertension^37^. This may in part be due to a change in the reporting of hypertension in 2019, from 140 mmHg systolic to 130 mmHg systolic^33,37^. The American Heart Association (AHA) statistics show this recalculated prevalence to be 46%^37^. Since the majority of our EHR data was from before 2019, our calculation of 32.1% is consistent with earlier estimates (Fig. 3). We did not distinguish between controlled- or uncontrolled-hypertension, and we accepted any reported hypertension for inclusion (Table 1). We also did not require multiple diagnoses/medical record entries or evidence of medication for inclusion. The higher prevalence of a history of hypertension in patients with dementia (> 80%), noticed across all SIRE categories, supports the view that hypertension is a risk factor for dementia^1^.

The national prevalence of T2D is approximately 9% according to the CDC^34^. We calculated our prevalence rate to be 13.9%, which is higher than the national average, but may reflect a slightly higher age of our test population (40–80 vs. all ages). Similar to national statistics, we observe the highest T2D prevalence in Hispanic participants, followed by Black and White participants (19.8, 16.3, 10.7%, respectively), although our rates are higher than the national averages (12.5, 11.7, 7.5%, respectively). This may represent a more engaged participant population compared to participants with hypertension or dementia. Again, we focused on a primary diagnosis of T2D for inclusion in our study, and we did not assess for markers of aggressive diabetes, such as retinopathy or neuropathies, nor did we assess for diabetes medications. The prevalence of dementia in the AoU participant cohort is an order of magnitude lower than national statistics (Table 2).

Patients with dementia represent a vulnerable patient population due to cognitive decline. The low number of dementia diagnoses seen for the AoU cohort may reflect concern for such vulnerable participants, or lack of strategies to engage such participants. Clearly, informed consent of participants with dementias and reduced capacity for understanding the program present significant recruitment challenges. We did not stratify dementia into its many forms (such as Alzheimer’s disease, Lewy body dementia, fronto-temporal dementia, vascular dementia etc.). Recent studies suggest a difference in the prevalence of hypertension in patients with vascular dementia vs. Alzheimer’s disease^13,20^. Alzheimer’s disease was associated with lower blood pressure, whereas vascular dementia was strongly correlated with hypertension. We did not consider severity of the condition, or other known lifestyle risks such as head trauma, family history, smoking^1,8^. However, our data show a strong influence of a past-history of hypertension with dementia prevalence (Fig. 4). The prevalence of dementia is much lower in the AoU cohort compared to national estimates. We also observe strong associatons of hypertension with dementia for all SIRE categories, but only observe an association of T2D with dementia for Black and Hispanic participants. Much of the association of T2D may be accounted for due to concurrent hypertension. The consistent influence of hypertension suggests this may be a modifiable factor for dementia. We did not look at additional factors, and one potential confounding factor could be socioeconomic status and education levels. Interestingly, these factors are associated with higher prevalence of diabetes and hypertension, and therefore associated with increased risk of dementia. Clearly, further work is needed to separate out these issues.

The AoU Research Program is a longitudinal study of an engaged population designed to reflect the population of the US^32^, and as such there may be some study design limitations and biases. The sample population is varied across the country, depending on the engagement and recruitment strategy of a center making selection bias a challenge. The AoU program has made including minority and underrepresented communities a priority for their participant enrollment, and the demographic data reflect this. Although we noticed a strong drop-off in the numbers of elderly participants, and especially non-White elderly participants (Fig. 2).

Nearly half of the AoU participants did not have EHR for our study. One of the many challenges of extracting data from medical records is the possibility of inaccurate or missing records and diagnoses. The different prevalence of dementia, T2D, and hypertension in our study cohort compared to national statistics suggest potential misdiagnosis of these conditions, and in the case of patients with dementia, they are excluded from study (see “Methods”). We used consensus diagnosis to incorporate multiple potential diagnoses into one term for dementia, hypertension, and T2D, and did not cross verify with medications or repeat diagnoses. Dementia, and Alzheimer’s in particular, is challenging to diagnose, using multiple psychological tests, PET scanning, CSF biomarkers, and post-mortem brain examination/histology^28,38,39^. Such data are not yet available in the AoU Researcher Workbench.

There is an increase in prevalence of dementia in older age groups in the AoU cohort, and a strong association of hypertension in participants with reported dementia. These findings suggests that controlling hypertension may reduce the prevalence of dementia later in life. Clearly, dementia and Alzheimer’s disease are complex disorders with multiple modifying factors, such as socioeconomic status, smoking, and familial history, but our analysis aligns with the WHO guidance describing hypertension as a modifiable factor for dementia, and supports the investigation of hypertension controlling approaches for reducing dementia.

## Methods

### Participant consent and IRB review

All methods were carried out in accordance with local guidelines and regulations. The data project and experimental protocols were approved by the AoU Institutional Review Board (#2016–05-TN-Master). This work was performed as part of a Data Demonstration Project for the AoU Research Program ^36^. AoU Demonstration Projects were designed to describe the cohort, replicate previous findings for validation, and avoid novel discovery. The initial release of data and tools used in this work was published recently^40^. Results reported here comply with the AoU Data and Statistics Dissemination Policy disallowing disclosure of group counts under 20.

Informed consent was obtained from all AoU participants. Participants were recruited to the study through a variety of engagement activities^32^. AoU inclusion criteria include adults 18 and older, with the legal authority and decisional capacity to consent, and currently residing in the US or a territory of the US. Exclusions include people age lower than 18, and vulnerable populations (prisoners and people without the capacity to give consent). Details on informed consent and recruitment inclusion criteria are available online at (https://allofus.nih.gov/sites/default/files/aou_operational_protocol_v1.7_mar_2018.pdf).

### AoU participant cohort construction

Data elements were extracted from the AoU Researcher Workbench. Documentation on the AoU Curated Data Repository (CDR) is available in the Registered Tier CDR Data Dictionary (https://www.researchallofus.org/methods). Participants included in this CDR (version R2019Q4R3) were enrolled as part of the NIH AoU Research Program between 2018–2019 (cut off 09/01/2019); of 223,921 participants, 156,648 were determined to have EHR data.

The cohort and data set construction is described in the Researcher Workbench workspace Dementia-Hypertension-Diabetes2, **Notebook: Project026 DataPrep** (Fig. 1: the scripts used are available in the Researcher Workbench). We used the Dataset Builder to create a dataset of patients with dementia, hypertension, and T2D, based on EHR record ICD9/ ICD10 codes, at any time during the participants’ history, by collapsing similar conditions into three main condition groupings (see Supplementary Table 1). We created a data frame with participant demographic data and the three consensus conditions, which was used for subsequent analysis (Fig. 1).

### Statistical analysis

The cohort and data set construction is described in the Researcher Workbench workspace Dementia-Hypertension-Diabetes2, Notebook: Project026Analysis_1 (see Researcher workbook for commands). All other data and statistical analysis were performed using R running on the Jupyter notebook on the *All of Us* server.

Two-way contingency tables were analyzed using Pearson's Chi-square test with Yates' continuity correction, and multi-proportional tables were analyzed using the Cochran-Mantel–Haenszel test using R (Version 4.0.2). Significance was accepted at p < 0.05. A multivariable logistic regression model was used to associate dementia case–control status (outcome) with hypertension, T2D, age decade, sex, and SIRE group (predictors). The model was used to calculate odds ratios and 95% confidence intervals for each predictor. The R package ‘dominanceanalysis’ v1.3.0 was used to rank the relative importance of predictors in the multivariable logistic regression model^41,42^.

## Supplementary Information

Supplementary Information.

## Data Availability

The datasets used and/or analyzed during the current study are available to US based researchers at the NIH All of Us researcher workbench (https://www.researchallofus.org/data-tools/workbench/). At time of press this data is only accessible to US based researchers, with institutional access to the researcher workbench. Code/ R commands used for analysis is available on the research workbench, or from the corresponding author on reasonable request.

## References

[1] Alzheimer’s disease facts and figures (2020). Alzheimers Dement.

[2] Ballard C (2011). Alzheimer’s disease. Lancet.

[3] Hardy J, De Strooper B (2017). Alzheimer’s disease: Where next for anti-amyloid therapies?. Brain.

[4] De Strooper B, Karran E (2016). The cellular phase of Alzheimer’s disease. Cell.

[5] Giacobini E, Gold G (2013). Alzheimer disease therapy–moving from amyloid-beta to tau. Nat. Rev. Neurol..

[6] Baumgart M (2015). Summary of the evidence on modifiable risk factors for cognitive decline and dementia: A population-based perspective. Alzheimers Dement..

[7] in *Risk Reduction of Cognitive Decline and Dementia: WHO Guidelines WHO Guidelines Approved by the Guidelines Review Committee* (2019). 31219687

[8] Livingston G (2020). Dementia prevention, intervention, and care: 2020 report of the Lancet Commission. Lancet.

[9] Mahase E (2019). WHO dementia guidelines highlight a lack of evidence for lifestyle interventions. BMJ.

[10] McGrath ER (2017). Blood pressure from mid- to late life and risk of incident dementia. Neurology.

[11] Skoog I (1996). 15-year longitudinal study of blood pressure and dementia. Lancet.

[12] Lennon MJ, Makkar SR, Crawford JD, Sachdev PS (2019). Midlife hypertension and Alzheimer’s disease: A systematic review and meta-analysis. J. Alzheimers Dis..

[13] Javanshiri K (2018). Atherosclerosis, hypertension, and diabetes in Alzheimer’s disease, vascular dementia, and mixed dementia: Prevalence and presentation. J. Alzheimers Dis..

[14] Abell JG (2018). Association between systolic blood pressure and dementia in the Whitehall II cohort study: Role of age, duration and threshold used to define hypertension. Eur. Heart J..

[15] Iadecola C, Gottesman RF (2018). Cerebrovascular alterations in Alzheimer disease. Circ. Res..

[16] Romay MC, Toro C, Iruela-Arispe ML (2019). Emerging molecular mechanisms of vascular dementia. Curr. Opin. Hematol..

[17] Chatterjee S (2016). Type 2 diabetes as a risk factor for dementia in women compared with men: A pooled analysis of 2.3 million people comprising more than 100,000 cases of dementia. Diabetes Care.

[18] McMillan JM, Mele BS, Hogan DB, Leung AA (2018). Impact of pharmacological treatment of diabetes mellitus on dementia risk: Systematic review and meta-analysis. BMJ Open Diabetes Res. Care.

[19] Abner EL (2016). Diabetes is associated with cerebrovascular but not Alzheimer’s disease neuropathology. Alzheimers Dement..

[20] Javanshiri K, Haglund M, Englund E (2019). Cardiovascular disease, diabetes mellitus, and hypertension in Lewy body disease: A comparison with other dementia disorders. J. Alzheimers Dis..

[21] Biessels GJ, Despa F (2018). Cognitive decline and dementia in diabetes mellitus: Mechanisms and clinical implications. Nat. Rev. Endocrinol..

[22] Burillo J (2021). Insulin resistance and diabetes mellitus in Alzheimer’s disease. Cells.

[23] Baglietto-Vargas D, Shi J, Yaeger DM, Ager R, LaFerla FM (2016). Diabetes and Alzheimer’s disease crosstalk. Neurosci. Biobehav. Rev..

[24] Ng TP (2014). Long-term metformin usage and cognitive function among older adults with diabetes. J. Alzheimers Dis..

[25] Caberlotto L (2019). Cross-disease analysis of Alzheimer’s disease and type-2 Diabetes highlights the role of autophagy in the pathophysiology of two highly comorbid diseases. Sci. Rep..

[26] Kandimalla R, Thirumala V, Reddy PH (2017). Is Alzheimer’s disease a type 3 diabetes? A critical appraisal. Biochim. Biophys. Acta Mol. Basis Dis..

[27] Sims-Robinson C, Kim B, Rosko A, Feldman EL (2010). How does diabetes accelerate Alzheimer disease pathology?. Nat. Rev. Neurol..

[28] Dilworth-Anderson P (2008). Diagnosis and assessment of Alzheimer’s disease in diverse populations. Alzheimers Dement..

[29] Perkins P (1997). Incidence and prevalence of dementia in a multiethnic cohort of municipal retirees. Neurology.

[30] Steenland K, Goldstein FC, Levey A, Wharton W (2016). A meta-analysis of Alzheimer’s disease incidence and prevalence comparing African-Americans and Caucasians. J. Alzheimers Dis..

[31] Haan MN (2003). Prevalence of dementia in older latinos: The influence of type 2 diabetes mellitus, stroke and genetic factors. J. Am. Geriatr. Soc..

[32] All of Us Research Program, I. *et al.* The "All of Us" Research Program. *N Engl J Med* **381**, 668-676, doi:10.1056/NEJMsr1809937 (2019).

[33] Whelton PK (2018). 2017 ACC/AHA/AAPA/ABC/ACPM/AGS/APhA/ASH/ASPC/NMA/PCNA guideline for the prevention, detection, evaluation, and management of high blood pressure in adults: A report of the American College of Cardiology/American Heart Association task force on clinical practice guidelines. Hypertension.

[34] (ed CDC) (2020).

[35] Hertz RP, Unger AN, Cornell JA, Saunders E (2005). Racial disparities in hypertension prevalence, awareness and management. Arch. Intern. Med..

[36] Denny JC, Devaney SA, Gebo KA (2019). The, “All of Us” research program reply. N. Engl. J. Med..

[37] Benjamin EJ (2018). Heart disease and stroke statistics-2018 update: A report from the American heart association. Circulation.

[38] Olsson B (2016). CSF and blood biomarkers for the diagnosis of Alzheimer’s disease: A systematic review and meta-analysis. Lancet Neurol..

[39] Sheikh-Bahaei N, Sajjadi SA, Pierce AL (2017). Current role for biomarkers in clinical diagnosis of Alzheimer disease and frontotemporal dementia. Curr. Treat Options Neurol..

[40] Ramirez AH (2020). The All of Us research program: Data quality, utility and diversity. medRxiv.

[41] Azen R, Budescu DV (2003). The dominance analysis approach for comparing predictors in multiple regression. Psychol. Methods.

[42] Budescu DV (1993). Dominance analysis: A new approach to the problem of relative importance of predictors in multiple regression. Psychol. Bull..

