# Supplementary material for: Investigation of hypertension and type 2 diabetes as risk factors for dementia in the All of Us cohort
Source: Sci Rep. 2022 Nov 17;12:19797. doi: 10.1038/s41598-022-23353-z (PMC9672061; doi:10.1038/s41598-022-23353-z)
Supplement: Supplementary file 1 — Supplementary Information. [file 41598_2022_23353_MOESM1_ESM.pdf]

Supplementary information Nagar et al

Supplementary data 1 Additional modeling data.

1. Adjusted model

Dementia ~ Hypertension + Type 2 Diabetes + Age + Sex + Race/ethnicity

Variable		Estimate	Std. Error	z value	Pr(>|z|)
(Intercept)		-8.2210	0.2652	-30.9970	5.92E-211
Hypertension		1.7778	0.1221	14.5619	4.91E-48
Type 2 diabetes		0.6737	0.0969	6.9512	3.62E-12
Age:	50-59	0.6470	0.2732	2.3684	1.79E-02
	60-69	1.7046	0.2533	6.7309	1.69E-11
	70+	2.2163	0.2548	8.6971	3.40E-18
Sex:	Female	0.0323	0.0896	0.3608	7.18E-01
SIRE:	Black or African American	-0.1241	0.1201	-1.0333	3.01E-01
	Hispanic	0.3560	0.1157	3.0780	2.08E-03
	Other	0.0234	0.1939	0.1205	9.04E-01

2. Interaction model

Dementia ~ Hypertension + Type 2 Diabetes + Age + Sex + Race/ethnicity + Hypertension * Race/ethnicity + Type 2 Diabetes * Race/ethnicity

Variable	Estimate	Std. Error	z value	Pr(>|z|)
(Intercept)	-7.9726	0.2729	-29.2121	1.36E-187
Hypertension	1.4856	0.1482	10.0269	1.16E-23
Type 2 diabetes	0.6864	0.1362	5.0400	4.66E-07
Age: 50-59	0.6324	0.2733	2.3142	2.07E-02
60-69	1.6819	0.2533	6.6391	3.16E-11
70+	2.1981	0.2547	8.6307	6.10E-18
Sex: Female	0.0178	0.0898	0.1984	8.43E-01
SIRE: Black or African American	-1.1469	0.3974	-2.8856	3.91E-03
Hispanic	-0.1382	0.2947	-0.4688	6.39E-01
Other	-0.3846	0.4648	-0.8274	4.08E-01
Hypertension * Black or African American	1.2555	0.4332	2.8980	3.75E-03
Hypertension * Hispanic	0.6856	0.3445	1.9905	4.65E-02
Hypertension * Other	0.3523	0.5545	0.6354	5.25E-01
Type 2 diabetes * Black or African American	-0.1208	0.2485	-0.4861	6.27E-01
Type 2 diabetes * Hispanic	-0.1188	0.2486	-0.4779	6.33E-01
Type 2 diabetes * Other	0.2665	0.4238	0.6288	5.29E-01

Supplementary Data 2 – Author lists

All of Us Research Program Demonstration Projects Subcommittee

First Name, Middle name/Initial	Last Name	Credentials	Department	Email	Institution	Location
Ashley	Able	PhD	Vanderbilt Institute for Clinical and Translational Research	ashley.a.able.1@vumc.org	Vanderbilt University Medical Center	2525 West End Ave, Nashville, TN, 37203
Eric	Boerwinkle	PhD	School of Public Health	eric.boerwinkle@uth.tmc.edu	The University of Texas Health Science Center at Houston	Houston, TX
Mine	Cicek	Ph.D.	Department of Laboratory Medicine and Pathology	cicek.mine@mayo.edu	Mayo Clinic	Rochester, MN
Cheryl R.	Clark	MD, ScD	Department of Medicine	crclark@bwh.harvard.edu	Brigham and Women’s Hospital	Boston, MA
Elizabeth	Cohn	RN, PhD	Hunter-Bellevue School of Nursing	elizabeth.cohn@hunter.cuny.edu	Hunter College City University of New York	New York, New York
Kelly	Gebo	MD, MPH	Bloomberg School of Public Health	kgebo@jhmi.edu	Johns Hopkins University School of Medicine	Bethesda, MD
Ashley	Green	MLIS	Vanderbilt Institute for Clinical and Translational Research	ashley.e.green@vumc.org	Vanderbilt University Medical Center	2525 West End Ave, Nashville, TN, 37203
Roxana	Loperena	PhD	Vanderbilt Institute for Clinical and Translational Research	roxana.loperena-cortes@vumc.org	Vanderbilt University Medical Center	2525 West End Ave, Nashville, TN. 37203
Kelsey	Mayo	PhD	Vanderbilt Institute for Clinical and Translational Research	kelsey.mayo@vumc.org	Vanderbilt University Medical Center	2525 West End Ave, Nashville, TN. 37203

Stephen	Mockrin	Ph.D.	*All of Us* Research Program	stephen.mockrin@nih.gov	National Institutes of Health, Leidos, Inc	5202 Presidents Ct, Suite 110, Frederick MD, 21703
Lucila	Ohno-Machado	MD, PhD	Department of Biomedical Informatics	lohnomachado@ucsd.edu	UCSD Health	La Jolla, CA
Andrea H.	Ramirez	MD, MS	Department of Medicine	andrea.h.ramirez@vumc.org	Vanderbilt University Medical Center	2525 West End Ave, Nashville, TN, 37203
Sheri	Schully	PhD	*All of Us* Research Program	schullys@mail.nih.gov	National Institutes of Health	Bethesda, MD

Condition	Condition Concepts
Consensus_Dementia	'Dementia; Alzheimer's disease
Consensus_Hypertension	'Essential hypertension: Elevated blood pressure: Malignant essential hypertension: Hypertensive disorder: Benign essential hypertension: Chronic hypertension complicating AND/OR reason for care during pregnancy Benign hypertension: Hypertensive urgency
Consensus_Diabetes	Type 2 diabetes mellitus in nonobese: Diabetes mellitus without complication: Diabetes mellitus Type 2 diabetes mellitus without complication: Hyperglycemia Type 2 diabetes mellitus Type II diabetes mellitus uncontrolled: Hypoglycemic state in diabetes: Hyperglycemia due to type 2 diabetes mellitus: Disorder due to type 2 diabetes mellitus: Type 2 diabetes mellitus well controlled: Insulin treated type 2 diabetes mellitus

Supplementary Table 1. Concept terms collapsed into consensus conditions.
